# Quality of Patient Information Websites About Congenital Heart Defects: Mixed-Methods Study of Perspectives Among Individuals With Experience of a Prenatal Diagnosis

**DOI:** 10.2196/ijmr.7844

**Published:** 2017-09-12

**Authors:** Tommy Carlsson, Ulla Melander Marttala, Barbro Wadensten, Gunnar Bergman, Ove Axelsson, Elisabet Mattsson

**Affiliations:** ^1^ Department of Women's and Children's Health Uppsala University Uppsala Sweden; ^2^ Department of Scandinavian Languages Uppsala University Uppsala Sweden; ^3^ Department of Public Health and Caring Sciences Uppsala University Uppsala Sweden; ^4^ Department of Women’s and Children’s Health Karolinska Institutet Stockholm Sweden; ^5^ Centre for Clinical Research Sörmland Uppsala University Eskilstuna Sweden; ^6^ Department of Health Care Sciences Ersta Sköndal Bräcke University College Stockholm Sweden

**Keywords:** congenital heart defects, consumer health information, information literacy, Internet, popular works, pregnancy, prenatal diagnosis

## Abstract

**Background:**

When a heart defect is prenatally diagnosed in the fetus, expectant parents experience a great need for information about various topics. After the diagnosis, the Web is used for supplemental information, and the scarcity of research calls attention to the need to explore patient information websites from the perspectives of the intended consumers.

**Objective:**

The overarching aim of this study was to explore the quality of Swedish patient information websites about congenital heart defects, from the perspectives of individuals with experience of a prenatal diagnosis of congenital heart defect in the fetus.

**Methods:**

This was a mixed-methods study of websites identified through systematic searches in the two most used Web-based search engines. Of the total 80 screened hits, 10 hits led to patient information websites about congenital heart defects. A quality assessment tool inspired by a previous study was used to evaluate each website’s appearance, details, relevance, suitability, information about treatment choices, and overall quality. Answers were given on a 5-point Likert scale, ranging from 1, representing the lowest score, to 5, representing the highest score. Each website was assessed individually by persons with experience of continued (n=4) and terminated (n=5) pregnancy following a prenatal diagnosis. Assessments were analyzed with Kendall’s coefficient of concordance W, Mann-Whitney *U* test, Friedman’s test, and a Wilcoxon-Nemenyi-McDonald-Thompson test. In addition, each assessor submitted written responses to open-ended questions in the quality assessment tool, and two joint focus group discussions were conducted with each group of assessors. The qualitative data were analyzed with inductive manifest content analysis.

**Results:**

Assessments represented a low score (median=2.0) for treatment choices and moderate scores (median=3.0) for appearance, details, relevance, suitability, and overall quality. No website had a median of the highest achievable score for any of the questions in the quality assessment tool. Medians of the lowest achievable score were found in questions about treatment choices (n=4 websites), details (n=2 websites), suitability (n=1 website), and overall quality (n=1 website). Websites had significantly different scores for appearance (*P*=.01), details (*P*<.001), relevance (*P*<.001), suitability (*P*<.001), treatment choices (*P*=.04), and overall quality (*P*<.001). The content analysis of the qualitative data generated six categories: (1) advertisements, (2) comprehensiveness, (3) design, (4) illustrations and pictures, (5) language, and (6) trustworthiness. Various issues with the included websites were highlighted, including the use of inappropriate advertisements, biased information, poor illustrations, complex language, and poor trustworthiness.

**Conclusions:**

From the perspectives of the intended consumers, patient information websites about congenital heart defects are, to a large extent, inadequate tools for supplemental information following a prenatal diagnosis. Health professionals should initiate discussions with patients about their intentions to use the Web, inform them about the varied quality in the Web-based landscape, and offer recommendations for appropriate Web-based sources.

## Introduction

### Background

Many countries around the world include obstetric ultrasound examinations as part of routine maternity care. One of the investigations possible with the ultrasound examination involves assessment of the anatomy of fetuses, with the purpose to detect or rule out fetal anomalies [1]. The most common types of fetal anomalies are congenital heart defects, defined as malformations of the heart and great vessels [2]. The incidence of congenital heart defects is approximately 8 in 1000 live births [2], with an incidence of 2.5 to 3.0 for the most severe, which require specialist cardiologic care [3]. Between the years 2000 and 2005, roughly 1 in 5 cases of all congenital heart defects were detected before birth [4]. However, the rates of prenatal diagnoses have increased in recent years [5-7], and studies report that over 40% of the most severe defects are prenatally diagnosed today [2,4,7].

Expectant parents view the obstetric ultrasound examination as an important step toward parenthood [8-10] but are often unprepared for adverse news [11-13] and experience an emotional crisis when faced with a prenatal diagnosis [11,14]. Depending on legislation and availability of induced abortion, the diagnosis may require a decision to continue or terminate the pregnancy. Expectant parents faced with the diagnosis experience this decision as difficult [11,15]. The decision involves various informational [11,16,17] considerations such as the process of induced abortion and what to expect of the postnatal situation if the pregnancy is continued [17]. Consequently, these persons experience a great need for information and highly value that which is offered during consultations with specialist health professionals [17,18]. However, the psychological distress experienced in connection to the diagnosis negatively influences comprehension and retention of the information offered [12,17], and research suggests that these individuals use the Web for supplemental information [16,17,19].

Patients view the Web as an important source of health-related information [20] and commonly use it for such purposes [20,21]. During pregnancy, women frequently use it to search for information about a variety of topics such as fetal development, pregnancy complications, and antenatal care [22]. As a large source of potentially highly accessible information [23,24], it holds promise to promote patient equity and empowerment [23,25-27]. However, studies indicate that consumers experience various difficulties when using the Web for health-related information [28,29]. Further complicating the situation, there is a lack of overarching systematic activities that aim to assess and control the quality of the available Web-based information [23], resulting in a risk of contact with information of poor quality. Combined, these issues call attention to the disorganized state of the Web [24]. The informational difficulties [11,15-17] and ethical dilemmas [30] related to decision making regarding whether to continue or terminate the pregnancy highlight the need to explore the relevance, level of details, suitability, and information about treatment choices in patient information websites. Moreover, research indicates that users of Web-based information place high importance on website appearance [31-33], raising questions concerning whether the design of these websites correspond to the preferences of the intended consumers.

As a step toward ensuring high-quality patient information materials, research suggests a need for developers to involve the intended consumers in the stages of production [34]. However, studies investigating the quality of Web-based information often use researchers or health professionals as assessors, raising questions about its applicability in clinical settings. In light of the identified differences in perspectives between researchers, health professionals, and patients [35,36], more research that employs new methods to investigate the perspectives of the actual consumers of the information is needed. No study has yet been published that explores consumers’ perspectives on websites about congenital heart defects.

### Objectives

The overall aim of this study was to explore the quality of patient information websites about congenital heart defects, from the perspectives of individuals with personal experience of a prenatal diagnosis of congenital heart defect in the fetus. Specifically, we set out to address the following research questions:

- How do individuals with personal experience of a prenatal diagnosis of congenital heart defect in the fetus assess patient information websites about congenital heart defects with regard to appearance, details, relevance, suitability, information about treatment choices, and overall quality?
- Are there any differences in the aforementioned assessments between (1) assessors with continued and terminated pregnancy, and (2) websites about congenital heart defects?
- What perspectives do individuals with experience of a prenatal diagnosis bring up when accessing these websites?

## Methods

### Study Context

In Sweden, all expectant parents are offered a routine ultrasound examination at approximately 18 weeks of gestation. One of the purposes of the examination is to assess the fetal anatomy, to detect or rule out possible fetal anomalies. Swedish law permits termination of pregnancy up to 18 completed weeks of gestation. At later gestations, approval must be granted by the National Board of Health and Welfare. Few pregnancies are terminated after 22 completed weeks.

### Study Design

Through mixed-methods design with quantitative and qualitative approaches, we aspired to utilize the strengths and to offset the weaknesses of each approach [37]. Mixed-methods designs are presented as promising strategies to optimize assessments of Web-based health-related information [38].

### Patient Information Websites

**Figure 1:**
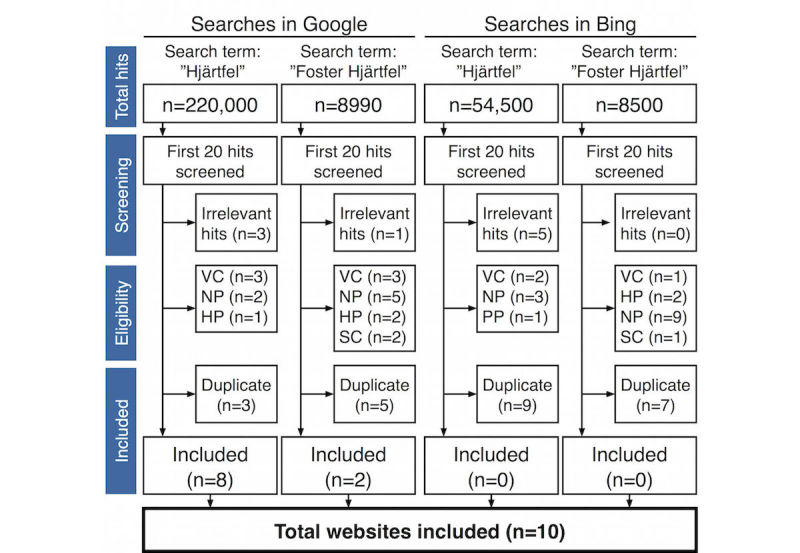
Search procedure to identify Swedish patient information websites about congenital heart defects. VC=virtual community or blog; HP=website specifically produced for health professionals; NP=newspaper website; PP=password-protected website; SC=scientific paper.

The two most popular search engines on the Web, Google and Bing [39], were used to search for Swedish patient information websites about congenital heart defects. Swedish search terms for *heart defect* (*hjärtfel*) and *fetus heart defect* (*foster hjärtfel*) were used in searches during May 2015. In total, this resulted in 291,990 hits. We performed the searches in the incognito mode of our Web browser, to minimize influence from previous search patterns. The first 20 hits of each search were screened for inclusion by the first author. In total, 80 hits were screened; nine hits were deemed irrelevant, as they did not include any information about congenital heart defects. Relevant hits that included information about congenital heart defects leading to newspaper websites (n=19), communities or blogs (n=9), websites specifically produced for health professionals (n=5), scientific papers (n=3), and password-protected websites (n=1) were excluded. After correcting for duplicate hits (n=24), 10 Swedish patient information websites about congenital heart defects were included (Figure 1).

The first author decided which search terms to use. After the assessments, we asked the assessors, with experience of a prenatal diagnosis, which search terms they preferred to use when searching for patient information websites about congenital heart defects in connection to the diagnosis. The most commonly reported words in these search terms were *heart defect* (n=29 times mentioned) and *fetus* (n=11 times mentioned) (Multimedia Appendix 1).

The included websites were affiliated with charities and private organizations (n=3); government, hospitals, and clinics (n=3); independent information websites (n=2); and pharmaceutical companies (n=2). Of the included websites, 9 appeared in the first 10 hits of the searches, and 8 were identified in more than one of the searches (Multimedia Appendices 2 and 3).

### Assessors

Assessors were purposefully recruited [40] to strive for variation with regard to country of birth, educational level, and age. They were recruited from 2 units for fetal cardiology in Sweden. Through her clinical network, the last author was responsible for the recruitment of assessors with continued pregnancy. In total, 8 potential assessors were asked to participate; 2 declined because of fear of rekindled painful memories. When contacted for further information, 2 females declined because (1) she felt like it was enough with her partner participating in the study (n=1 potential assessor) and (2) lack of time (n=1 potential assessor). Thus, 2 females and 2 males with continued pregnancy, assessed the websites. The first author was responsible for the recruitment of persons with terminated pregnancy. Potential assessors were identified when they took part in a previous interview study that used consecutive clinical recruitment [17]. In total, 8 potential assessors were asked to participate; 1 male declined because of lack of interest. When contacted for further information, 1 female and 1 male declined because of lack of time. Thus, 3 females and 2 males with terminated pregnancy assessed the websites.

Assessors with continued pregnancy were parents of children with congenital heart defects who were 2 (n=2 assessors) and 3 (n=2 assessors) years old. Assessors’ ages ranged between 23 and 43 years. Regarding country of birth, 3 assessors with continued pregnancy were born outside Sweden, and all with terminated pregnancy were born in Sweden. Of the assessors, 2 had senior high school and 7 had university/college as highest educational level (Multimedia Appendix 4). Assessors with terminated pregnancy had undergone the procedure 20 (n=1 assessor), 24 (n=2 assessors), 27 (n=1 assessor), and 38 (n=1 assessor) weeks before assessments. Of the assessors, 4 with continued (n=2 assessors) and terminated (n=2 assessors) pregnancy were couples, and so experienced the same prenatal diagnosis. Assessors with continued pregnancy had experience of ventricular septal defect (n=2 assessors) or a combination of transposition of the great arteries, ventricular septal defect, and pulmonary stenosis (n=2 assessors). Assessors with terminated pregnancy had experience of atrioventricular septal defect with associated trisomy 21 (n=2 assessors), aortic stenosis (n=2 assessors), or a combination of Ebstein’s anomaly and multiple structural malformations (n=1 assessor).

### Data Collection

#### Quantitative Data Collection

During 2015, individual written assessments were performed by the assessors for each of the included website. A quality assessment tool was developed (Table 1), inspired by a previous study within fetal cardiology [41]. The tool included 6 questions regarding each website’s appearance, details, relevance, suitability, information about treatment choices, and overall quality. Answers were given on a 5-point Likert scale, ranging from 1, representing lowest score, to 5, representing the highest score. A score of 3 represented quality criterion partly fulfilled, neither unsuitable nor suitable, and moderate overall quality. With 9 assessments for each of the 10 websites, we received a total of 90 assessments for each question in the quality assessment tool. In total, 8 assessors completed the assessments during a 4-hour workshop and 1 assessor at his home. Assessors were instructed to assess each included website separately and to access the sections that they found relevant to the subject of congenital heart defects when faced with a prenatal diagnosis (irrespective of which heart defect they had experienced). Because we aimed to collect assessments grounded in the perspectives of the intended consumers of the included websites, we did not provide further specific instructions to the assessors.

#### Qualitative Data Collection

Each question in the quality assessment tool was supplemented by a written open-ended question asking whether the assessors had any opinions related to the specific question. Additionally, assessors were asked to leave written comments regarding their perspectives on the positive and negative aspects of each website. Two focus group discussions were held regarding perspectives on the included websites, one with assessors who continued (n=4 assessors) and one with assessors who terminated (n=5 assessors) the pregnancy. The discussions were performed with the aid of a computer connected to a projector and were digitally recorded with audiovisual screen recordings. The first author moderated the discussions, and the second author attended the sessions as an observer. The websites were accessed one at a time, and assessors were encouraged to freely discuss aspects perceived as relevant to them. The discussions lasted 55 min for the assessors with continued pregnancy and 46 min for the assessors with terminated pregnancy. The first author transcribed the recordings verbatim.

**Table 1:** Quality assessment tool.

Question	Scale
Is the appearance appropriate for the target audience?	1 (no); 2; 3 (partly); 4; 5 (yes)
Is the level of detail appropriate for the target audience?	1 (no); 2; 3 (partly); 4; 5 (yes)
Is the content of the website relevant?	1 (no); 2; 3 (partly); 4; 5 (yes)
How suitable is the website as a source of information following a detection of congenital heart defect in the fetus?	1 (very unsuitable); 2; 3 (neither unsuitable nor suitable); 4; 5 (very suitable)
Is it clear that more than one treatment choice exists?	1 (no); 2; 3 (partly); 4; 5 (yes)
How is the overall quality of the website as a source of information following a detection of congenital heart defect in the fetus?	1 (low); 2; 3 (moderate); 4; 5 (high)

### Data Analysis

#### Quantitative Data Analysis

Quantitative data were analyzed with R version 3.2.2. (R Foundation for Statistical Computing, Austria). Kendall’s coefficient of concordance W was used to determine interrater reliability, with W ≥.21 representing fair, W ≥.41 moderate, W ≥.61 substantial, and W ≥.81 almost perfect concordance [42]. The median scores of all 10 included websites were calculated for each assessor, and the Mann-Whitney *U* test was used to compare these with the groups of assessors who continued and terminated the pregnancy. Friedman’s test was used to compare the scores of the websites, and the Wilcoxon- Nemenyi-McDonald-Thompson test was used as a posthoc test to investigate possible differences between the scores of specific websites. *P* values of <.05 were considered statistically significant.

#### Qualitative Data Analysis

Qualitative data were analyzed with Nvivo for Mac version 11.4.0. (QRS International Pty Ltd., Australia). The written responses to the open-ended questions and the transcripts of the focus group discussions were analyzed with inductive qualitative manifest content analysis, inspired by the outline presented by Graneheim and Lundman [43]. The materials were read repeatedly to gain an overall understanding. Meaning units were identified, defined as words, sentences or paragraphs containing aspects related to each other through a common content and context. Meaning units were condensed, that is, shortened without losing content and context. Condensed meaning units were each assigned descriptive codes, functioning as labels of the content. Codes were structured into categories, defined as collections of codes that shared a similar commonality with internally homogenous and externally heterogeneous manifest content, that is, the visible content described with as little interpretation as possible [43].

## Results

### Quantitative Results

**Figure 2:**
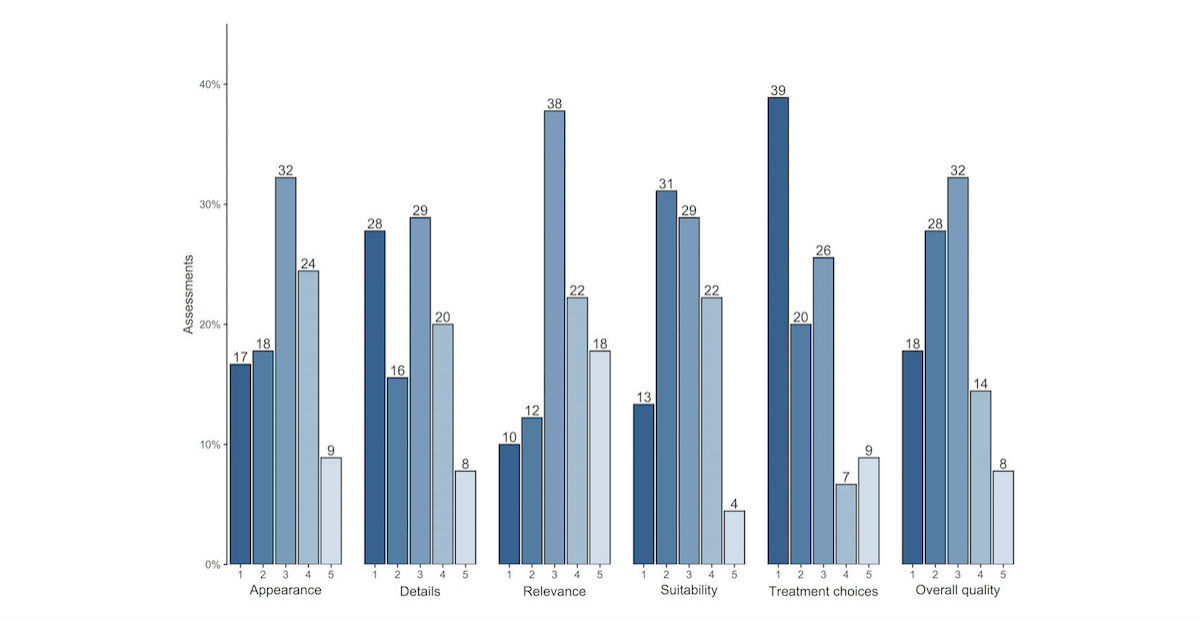
Distribution (percentage) of all assessment scores (n=90) for each question in the quality assessment tool. 1=low quality, 5=high quality.

Inspecting all of the assessments (n=90), the highest proportion of the lowest score (1) was found for treatment choices (n=35, 39% of total assessment scores). By contrast, the highest proportion of the highest score (5) was found for relevance (n=16, 18% of total assessment scores); see Figure 2.

**Figure 3:**
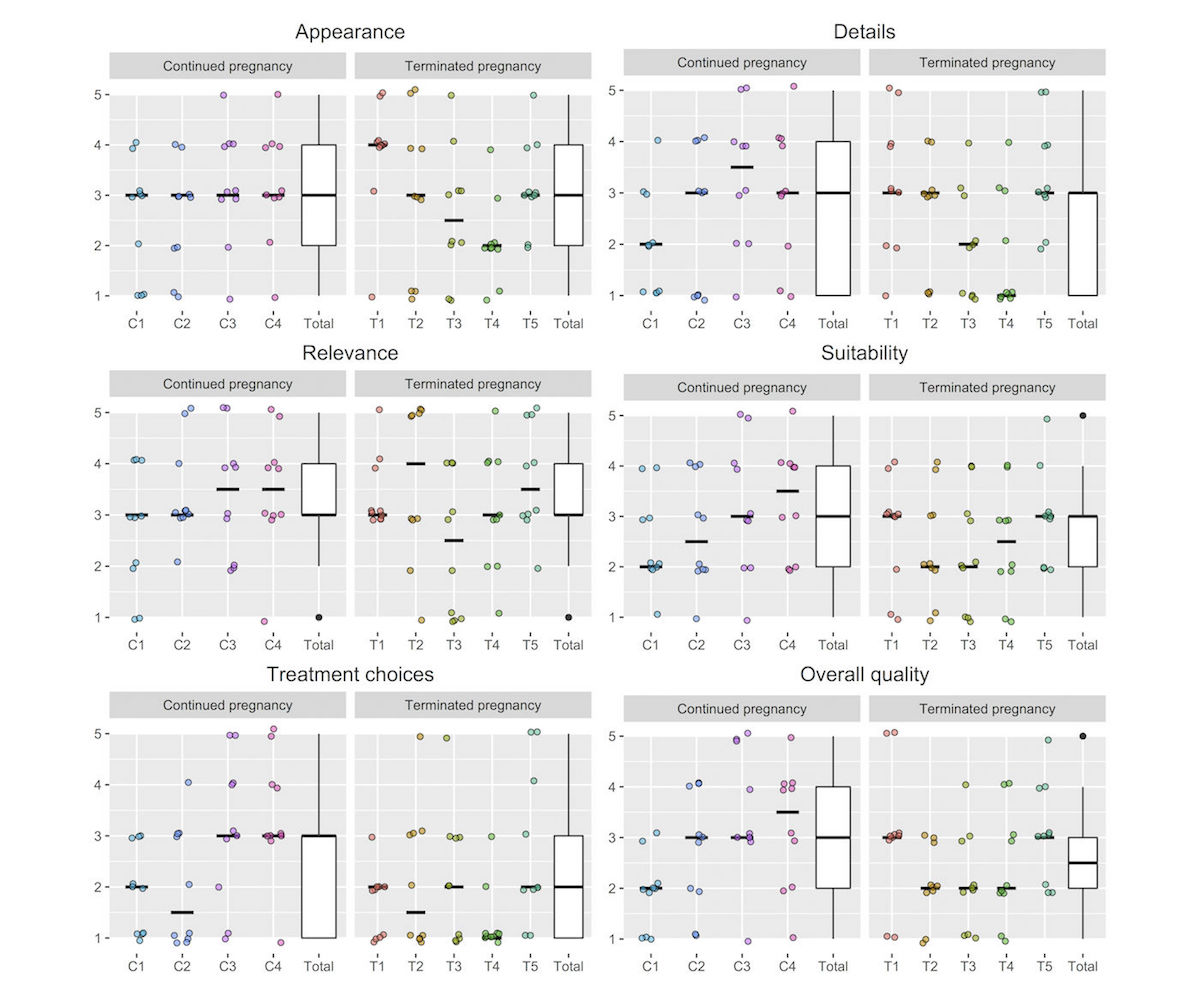
Distributions and medians (horizontal lines in dotplots) of the assessment scores from each of the assessors with continued (n=4) and terminated (n=5) pregnancy. 1=low quality; 5=high quality; C=continued pregnancy; T=terminated pregnancy.

For all assessors, the interrater reliability ranged between 0.18 and 0.40 for the 6 questions in the quality assessment tool. Interrater reliability ranged between 0.36 and 0.66 for assessors with continued pregnancy and between 0.29 and 0.42 for assessors with terminated pregnancy. With the exception of treatment choices (median=2.0), all questions had a median total score of 3.0, representing moderate quality (Table 2). There were no significant differences between median scores of the assessments from assessors with continued and terminated pregnancy. Figure 3 presents the distributions of the assessment scores among the assessors with continued and terminated pregnancy.

**Figure 4:**
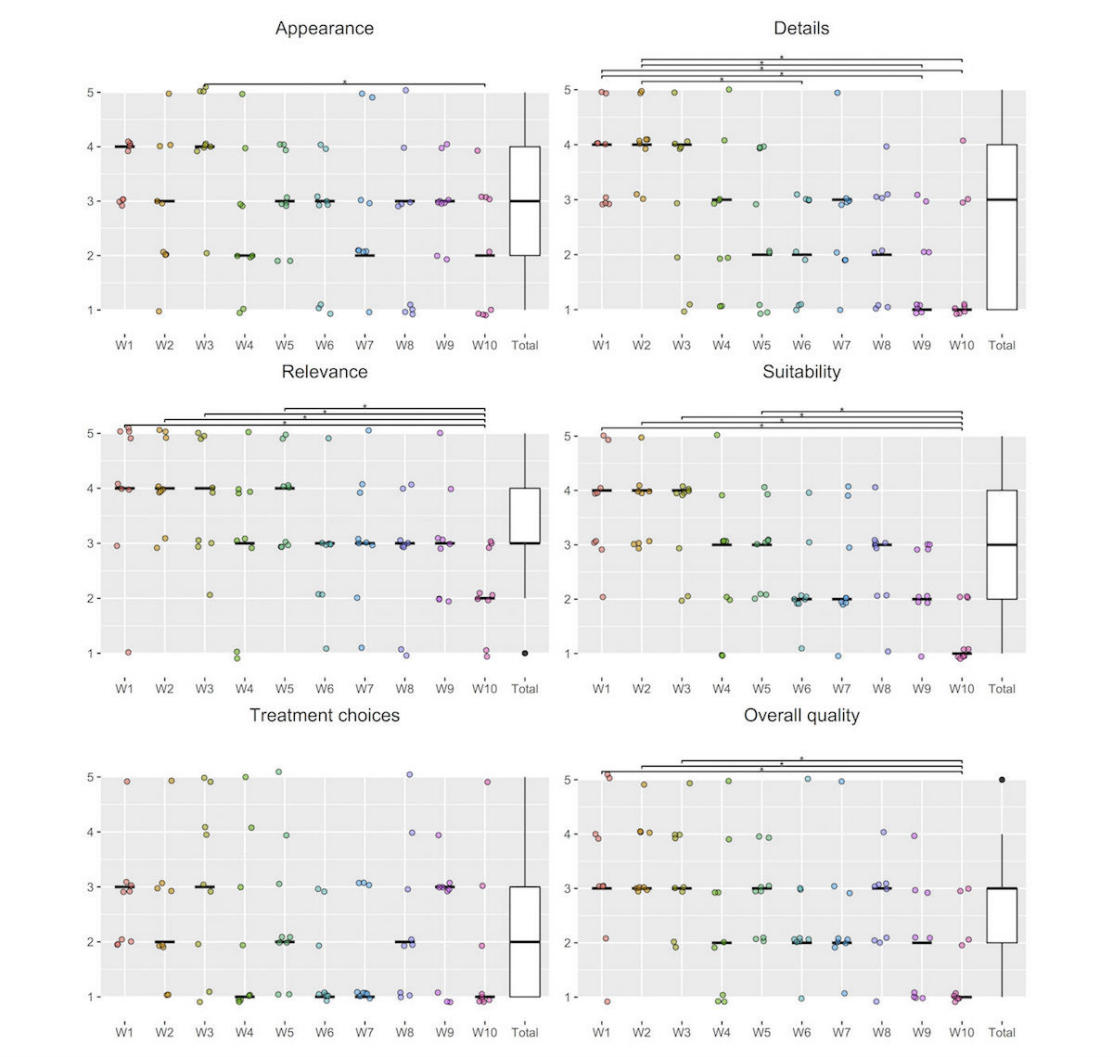
Distributions and medians (horizontal lines in dotplots) of the assessment scores for each of the websites included (n=10), with significant differences between websites indicated with brackets above plots. 1=low quality; 5=high quality; W=website; *P<.05.

Figure 4 presents the distributions of the assessment scores for each of the included websites. No website had a median score of 5.0, representing high quality, for any of the questions in the quality assessment tool. Median scores of 1.0, representing low quality, were found in questions about treatment choices (n=4 websites), details (n=2 websites), suitability (n=1 website), and overall quality (n=1 website). Website 10 had median scores of 1.0 for 4 of the 6 questions.

**Table 2:** Interrater reliability assessment scores for the questions in the quality assessment tool, with distributions of the assessment from assessors with continued pregnancy (n=40 assessments) and terminated pregnancy (n=50 assessments).

Questions	Continued pregnancy			Terminated pregnancy			Total		
	Interrater reliability^a^	Median	Interquartile range	Interrater reliability^a^	Median	Interquartile range	Interrater reliability^a^	Median	Interquartile range
Appearance	0.36	3.0	2	0.34	3.0	2	0.23	3.0	2
Details	0.60	3.0	3	0.42	3.0	2	0.38	3.0	3
Relevance	0.54	3.0	1	0.32	3.0	1	0.31	3.0	1
Suitability	0.66	3.0	2	0.42	3.0	1	0.40	3.0	2
Treatment choices	0.43	3.0	2	0.32	2.0	2	0.18	2.0	2
Overall quality	0.57	3.0	2	0.29	2.5	1	0.31	3.0	1

^a^Kendall’s coefficient of concordance W.

#### Appearance

Friedman’s test revealed significant differences between websites (χ^2^_9_=20.9, *P*=.01). Post hoc revealed significantly lower score for website 10 than website 3 (*R*=43.5, *P*=.008).

#### Details

Friedman’s test revealed significant differences between websites (χ^2^_9_=33.7, *P*<.001). Post hoc revealed a significantly lower score for website 6 than websites 1 (*R*=38.0, *P*=.05) and 2 (*R*=39.0, *P*=.04), for website 9 than websites 1 (*R*=45.5, *P*<.001) and 2 (*R*=46.5, *P*=.002), and for website 10 than websites 1 (*R*=45.0, *P*=.005) and 2 (*R*=46, *P*=.003).

#### Relevance

Friedman’s test revealed significant differences between websites (χ^2^_9_=28.9, *P*<.001). Post hoc revealed a significantly lower score for website 10 than websites 1 (*R*=44.5, *P*=.004), 2 (*R*=47.5, *P*=.002), 3 (*R*=39.5, *P*=.02), and 5 (*R*=37.5, *P*=.045).

#### Suitability

Friedman’s test revealed significant differences between websites (χ^2^_9_=35.7, *P*<.001). Post hoc revealed significantly lower score for website 10 than websites 1 (*R*=54.5, *P*<.001), 3 (*R*=51.5, *P*<.001), and 5 (*R*=39.5, *P*<.04).

#### Treatment Choices

Friedman’s test revealed significant differences between websites (χ^2^_9_=17.7, *P*=.04). Post hoc revealed no significant differences.

#### Overall Quality

Friedman’s test revealed significant differences between websites (χ^2^_9_=28.1, *P*<.001). Post hoc revealed significantly lower score for website 10 than websites 1 (*R*=39.5, *P*=.03), 2 (*R*=47.0, *P*<.002), and 3 (*R*=42.0, *P*<.01).

### Qualitative Results

Qualitative analysis generated six categories: (1) advertisements, (2) comprehensiveness, (3) design, (4) illustrations and pictures, (5) language, and (6) trustworthiness.

#### Advertisements

Advertisements made it difficult to recognize relevant information and were considered inappropriate in the context of prenatal diagnosis. When advertisements were used, the assessors regarded websites as less serious.

Children’s party [in an advertisement]! Not really the first thing that comes to mind…It was more like that disturbed me.

Male 3, continued pregnancy

#### Comprehensiveness

Overall, medical information about the normal cardiovascular system and specific heart defects were regarded as sufficient and of good quality. Statistics, as well as information about postnatal care and quality of life, were appreciated. However, assessors pointed out a lack of information about the postnatal situation for families and inclusion of irrelevant topics. Assessors in both groups appreciated stories of previous cases, considered these to provide realistic insights into postnatal situation. However, those with terminated pregnancy pointed out a lack of stories about previous cases that terminated the pregnancy.

It doesn’t say anything about how you would perform the surgery and nothing about other types of knowledge necessary about related subjects, how it is for the family...How will the child feel, how will it affect the parents.

Female 5, continued pregnancy

Information was considered belittling regarding the consequences of living with a severe heart defect, portraying an overly optimistic view of treatment and prognosis of severe defects. Balanced information about the alternatives to continue or terminate the pregnancy was considered lacking, and assessors with terminated pregnancy pointed out that there was insufficient information about induced abortions. The assessors observed that only 2 websites mentioned the possibility of terminating the pregnancy, but these were considered too simplistic and to lack relevant information about the actual process associated with induced abortion.

Doesn’t include anything about pregnancy termination. They kind of direct the reader toward “it’s just to keep going...”

Male 2, terminated pregnancy

#### Design

Clean interfaces were appreciated, with easy navigation between different subjects covered in the information. However, disorganized structures resulted in many unnecessary clicks required to continue reading, which hindered information uptake.

I didn’t even see the information on that website because wherever you clicked you got to a new window and were transferred to another [website]. So for me, I did not even get as far as you did because I got so annoyed with the website.

Female 1, terminated pregnancy

#### Illustrations and Pictures

Clear and easy-to-read illustrations of the anatomy of the normal cardiovascular system and specific heart defects were appreciated and considered to promote information comprehension, and website quality was regarded as lower when websites lacked these tools. Assessors described other illustrations as irrelevant, unsuitable, and complex. Those with terminated pregnancy mentioned that use of pictures portraying children were inappropriate for the context of prenatal diagnosis. In contrast, those with continued pregnancy appreciated pictures of hospitalized children, as this was considered to promote realistic expectations of the postnatal situation.

One thing that I just don’t appreciate is when you go into these [websites], and you see a picture of [a child]...Because that becomes, how would our child have looked like [...] You don’t want that picture in the back of your head.

Male 2, terminated pregnancy

#### Language

Assessors appreciated websites that used understandable, easy-to-read language with comprehensible explanations and the option to read more by clicking on complex words. However, they described that websites used complex language with unnecessary medical terminology, which for them made it seem like the information was produced for health professionals rather than for laypersons.

Maybe a little too complicated in the text.

Male 3, continued pregnancy

#### Trustworthiness

Websites were considered trustworthy when information was updated, included references, and had specialist health professionals as authors of medical content. When one or more of these criteria were lacking, assessors raised suspicions about the information’s accuracy. Websites from pharmaceutical companies were considered to be less trustworthy because of the possibility that they were profit driven. One website contained a disclaimer that the content had not been professionally reviewed, which made assessors frustrated as they considered themselves incapable of judging the accuracy. Another website had not been updated in more than a decade, which was considered a serious shortcoming.

One thing I reacted upon when I sat and read, when you accessed certain websites, you saw last time updated in 2002, and then it didn’t feel very current.

Male 2, terminated pregnancy

## Discussion

### Principal Findings

This study aimed to explore the quality of patient information websites about congenital heart defects and to provide new insights into these sources from the perspectives of the intended consumers. To the extent of our knowledge, this study is the first to use individuals with experience of a prenatal diagnosis as assessors to explore the quality of information websites about congenital heart defects. Overall, the investigated quality criteria were quantitatively assessed as either partially or not fulfilled. Various issues were described during discussions, indicating a need for further improvement of Web-based information about congenital heart defects. The suitability was assessed as neither unsuitable nor suitable, and the assessors raised issues regarding untrustworthy information belittling regarding the consequences of living with a severe heart defect. All of these aspects are known risks with Web-based information [23].

Parents of children with congenital heart defects want information about appropriate websites in connection to a prenatal diagnosis [17,44], but a previous study suggests that few cardiologists provide these recommendations [45]. According to the assessments in this study, some websites are particularly poor choices as sources for supplemental Web-based information. For example, one website affiliated with a clinic, identified in three searches and placed high in the search rank, had significantly lower assessments across five of the six quality criteria. Combined, the quantitative and qualitative findings indicate a need for health professionals to acknowledge the existing quality deficits and varied quality of available websites and bring this subject up for discussion during consultations with individuals who plan to use it for supplemental information. These efforts are even more important in light of the reports that consumers of health information use suboptimal search strategies [28], are unsuccessful at finding satisfactory Web-based information about health-related topics [46], and rarely discuss Web-based information with health professionals [22]. However, we acknowledge that health professionals may lack the time needed to feel adequately updated about available Web-based sources [24,47] and experience challenges when consulting patients who read Web-based information [47,48]. To adequately meet these issues, there seems to be a need for systematic efforts from overarching institutions in charge of publishing such recommendations.

Our findings indicate a lack of Web-based information about pregnancy termination and a bias of information toward pregnancy continuation in websites about congenital heart defects. It is crucial that individuals faced with a prenatal diagnosis reach informed decisions on whether to continue or terminate the pregnancy. Research suggests that persons faced with a prenatal diagnosis need information about the option to terminate the pregnancy and use the Web for supplemental information [17]. However, few English websites about congenital heart defects contain this type of information, which the findings of this study also confirm for Swedish settings [41]. Preparatory information is a key aspect to quality abortion care [49], and previous studies report that patients have unanswered questions and feel unprepared for the abortion process [50-52]. It is of great concern that patients experience information about pregnancy termination as insufficient and high-blown, both from health professionals and from Web-based sources [17,50]. Moreover, previous studies indicate that websites about induced abortions contain inaccurate, misleading [53,54], unsuitable, and low-quality information [55], calling attention to the need for patients to cautiously use and interpret Web-based information about these topics. In light of our findings, health professionals must consider these aspects when consulting individuals who decide on pregnancy termination and must make efforts to ensure that patients come into contact with sufficient and relevant high-quality Web-based information. The findings illustrate the importance of coordinated informational approaches between units for fetal cardiology and fetal medicine.

The complex language and unnecessary medical terminology described by the assessors raises concerns regarding the readability of these sources. Readability levels above that recommended to the general, nonmedical audience have been observed among websites about noninvasive prenatal testing [56] and other pregnancy-related topics [57,58], echoing our findings. Illustrations are possible pedagogic tools to overcome readability issues and are especially important when offering information to individuals with low health literacy [59], that is, the degree to which individuals have the capacity to obtain, process, and understand health information [60]. Assessors appreciated clear and easy-to-read illustrations, described as beneficial to promote comprehension. However, they also pointed toward the use of complex and irrelevant illustrations, further illustrating the problems related to poor readability. In light of these findings, we recommend the use of supplemental high-quality illustrations when offering information about congenital heart defects, but efforts need to be made to ensure that they are understandable to the intended consumers and have a clear purpose. Moreover, website developers need to take steps to improve the readability of their Web-based information about congenital heart defects.

### Strengths and Limitations

The strength of this study is that we used mixed-methods design [38] to quantitatively investigate a set of variables while still remaining open to other perspectives with an inductive qualitative approach [61]. Assessors with personal experience assessed the websites, with experience of both continued and terminated pregnancy following a prenatal diagnosis. Typically, studies investigating the quality of information websites about reproductive health issues use a limited number of researchers or health professionals as assessors, frequently between 1 and 4 assessors [41,53,56,62-64]. In light of the identified differences in perspectives between researchers, health professionals, and patients [35,36], we argue that by using 9 laypersons with personal experience as assessors, our study provides insight that may more closely correspond to the views and preferences of the intended consumers of the patient information.

We used both female and male assessors with experience of either continuation or termination of pregnancy, which implies that the assessments include views and preferences of laypersons with various backgrounds and experiences. However, considering the variations in severity, prognosis, and treatment of the possible heart defects that can be prenatally diagnosed today [65,66], we acknowledge that the assessment of our assessors may differ from laypersons with experiences of other prenatal diagnoses. The interrater reliability ranged between 0.18 and 0.66, with higher coefficients of concordance for assessors who shared similar pregnancy outcome. This indicates at least fair to moderate concordance between the assessors used in this study [42]. Moreover, we did not use any validated instruments to assess the websites, as these include an extensive number of items [38]. We urge a need for development of less extensive alternatives, suitable for the purpose of quality assessment that uses laypersons as assessors.

We did not collect any information concerning the literacy levels of the assessors. It is possible that their levels were above or below the population norms among the intended consumers. Consequently, we cannot make any claims regarding their literacy levels and their representativeness. The results need to be interpreted with this in mind.

We wanted the focus group discussions to reflect the topics of importance for the assessors and not be colored by our preconceptions as researchers. Thus, no discussion guide was used to guide the discussions, and the assessors were encouraged to freely discuss aspects perceived as relevant to them. We argue that this approach was an appropriate way to stay close to the perspectives of the assessors.

Swedish websites were included, an important aspect to consider in the context of prenatal diagnosis, which differs in routines and legislation between countries. However, previous assessments of English websites with a health professional or researcher as an assessor indicate that the identified problems in this study transcend various settings and are similarly perceived by professionals and laypersons [41]. It is important to bear in mind that consumers rarely search beyond the first 10 hits in searches performed in search engines [28]. Consequently, we argue that strengths of this study are that we screened the first 20 hits and performed the searches in the most commonly used search engines [39]. Moreover, 24 of the 80 screened hits were duplicates, indicating that we achieved saturation in the inclusion of available websites. The first author chose the search terms. Consequently, it is possible that these do not fully correspond to those used by the intended consumers. However, after the data collection, we asked the assessors which search terms they preferred to use when searching for patient information websites about congenital heart defects in connection to the diagnosis. The answers confirmed the used search terms (Multimedia Appendix 1), indicating adequate representativeness in relation to those search terms that are used among the intended consumers. Moreover, the first author screened the hits and decided which hits to include as patient information websites. It is possible that laypersons would include other hits.

### Suggestions for Future Research

We encourage researchers to conduct studies that investigate how to improve the quality of Web-based information about congenital heart defects. Additionally, more research is needed to explore how individuals with low educational levels, individuals with low health literacy, and immigrants experience these websites.

### Recommendations for Website Developers

The results indicate that website developers must ensure that sufficient information about treatment choices are included, with balanced information concerning pregnancy termination and possible consequences of living with a severe heart defect. Efforts should be taken to include statistics, as well as information about postnatal care and quality of life. If previous cases of persons faced with prenatal diagnosis are presented, make sure to include examples of cases with continued and terminated pregnancies. Clean and easily navigated designs, without advertisements, are important aspects when developing and maintaining websites. Clear and easy-to-read illustrations of the anatomy of the normal cardiovascular system and specific heart defects are desired among the intended consumers and should be included in Web-based patient information. Details should always be provided concerning references, authorship, and date of production. Website developers should strive toward continuous updates of the website and clearly state when it has been updated.

### Conclusions

From the perspectives of the intended consumers, patient information websites about congenital heart defects do not fulfill quality criteria concerning information about treatment choices and only partly fulfill quality criteria concerning appearance, details, relevance, suitability, and overall quality. There are differences between existing websites, indicating variations in the Web-based landscape. The findings indicate that these websites include inappropriate advertisements, biased information, poor illustrations, complex language, and have issues with trustworthiness. Improvement is needed to meet the needs among the intended consumers for unbiased, complete, and trustworthy high-quality patient information websites. Combined, the findings suggest that existing patient information websites about congenital heart defects are, to a large extent, inadequate tools for supplemental information following a prenatal diagnosis.

When counseling expectant parents faced with a prenatal diagnosis of congenital heart defect in the fetus, we encourage health professionals to initiate discussions about their intentions to use the Web. Professionals should inform these persons about the varied quality in the Web-based landscape and offer recommendations for appropriate websites that include unbiased high-quality supplemental information. We encourage website developers to avoid (1) using inappropriate advertisements and pictures, (2) publishing information that the intended consumers may consider belittling regarding the impact of living with a severe heart defect, and (3) using complex illustrations without a clear purpose. Furthermore, we encourage them to (1) include information about pregnancy termination, (2) write information in a readable and understandable language, without the use of unnecessary medical terminology, (3) include stories from persons with lived experience of continuation and termination of pregnancy, (4) continuously update their websites with accurate information written by specialist health professionals, and (4) include a list of references.

## Appendix

Multimedia Appendix 1 Number of words reported more than one time in search terms preferred by the assessors.

Multimedia Appendix 2 Search rank order and outcome of the screened hits.

Multimedia Appendix 3 Affiliation, number of searches where identified, and search ranks for each included website.

Multimedia Appendix 4 Characteristics of the assessors with continued (n=4) and terminated (n=5) pregnancy.
