# Supplementary material for: Quality of Patient Information Websites About Congenital Heart Defects: Mixed-Methods Study of Perspectives Among Individuals With Experience of a Prenatal Diagnosis
Source: Interact J Med Res. 2017 Sep 12;6(2):e15. doi: 10.2196/ijmr.7844 (PMC5615220; doi:10.2196/ijmr.7844)
Supplement: Multimedia Appendix 1 [file ijmr_v6i2e15_app1.pdf]

Swedish word	Translated word	n
Hjärtfel	Heart defect	29
Foster	Fetus	11
Barn	Child	7
Hjärtebarn	Heart-child	5
[Specifika hjärtfel]	[Specific heart defects]	4
Överlevnad	Survival	4
Orsak	Cause	3
Hjärta	Heart	3
Down's syndrom	Down's syndrome	3
RUL	[Abbreviation for routine ultrasound screening]	2
Medfödda	Congenital	2
Livslängd	Lifespan	2
Leva	Live	2
Hjärtmissbildning	Heart malformation	2
Fostervattentest	Amniotic fluid test	2
Antal operationer	Number of surgeries	2
Risk	Risk	2
