# Supplementary figures and images for: Quality of Patient Information Websites About Congenital Heart Defects: Mixed-Methods Study of Perspectives Among Individuals With Experience of a Prenatal Diagnosis
Source: Interact J Med Res. 2017 Sep 12;6(2):e15. doi: 10.2196/ijmr.7844 (PMC5615220; doi:10.2196/ijmr.7844)

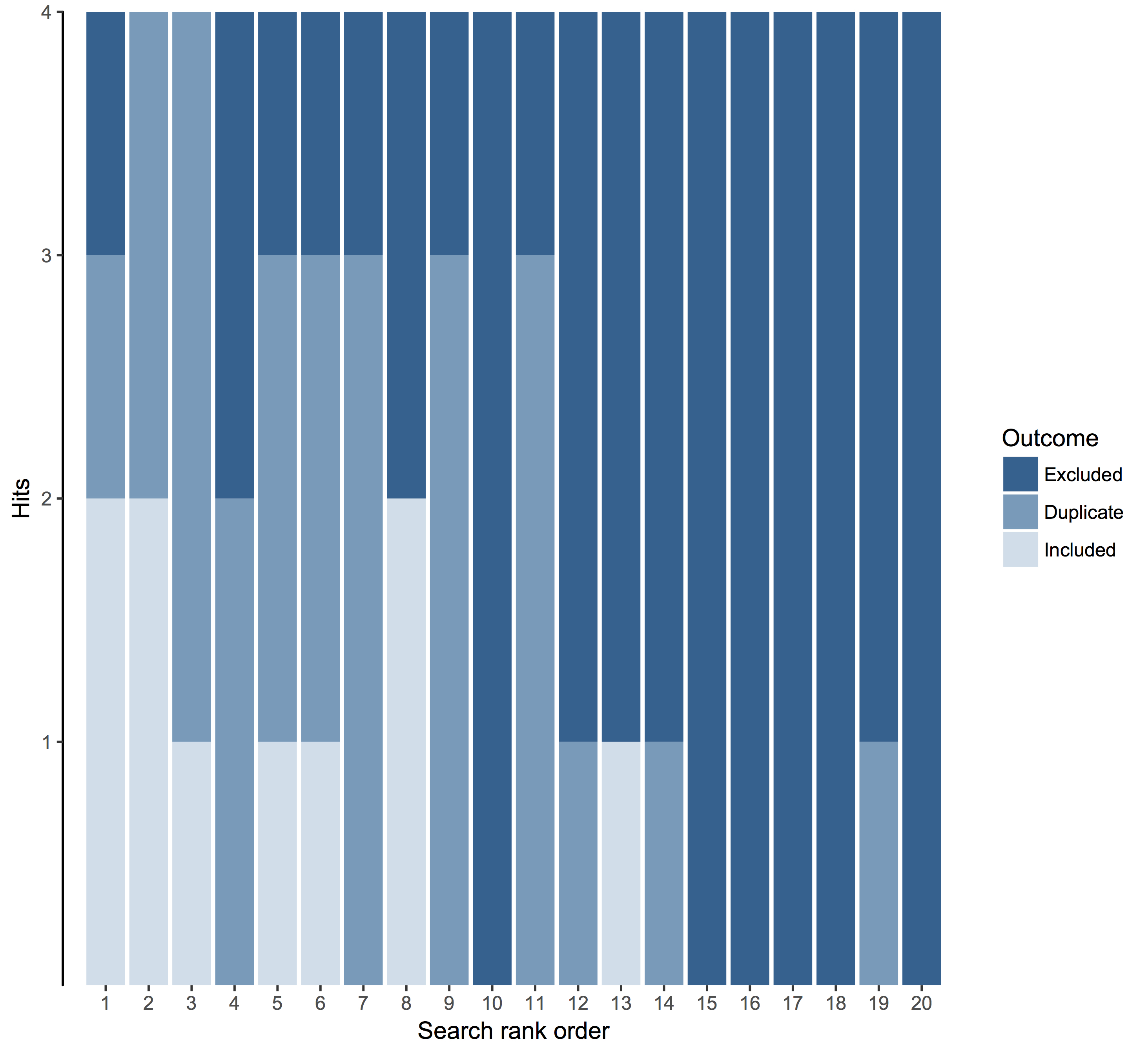

Supplement: Multimedia Appendix 2 [file ijmr_v6i2e15_app2.jpg]
