# Supplementary material for: Quality of Patient Information Websites About Congenital Heart Defects: Mixed-Methods Study of Perspectives Among Individuals With Experience of a Prenatal Diagnosis
Source: Interact J Med Res. 2017 Sep 12;6(2):e15. doi: 10.2196/ijmr.7844 (PMC5615220; doi:10.2196/ijmr.7844)
Supplement: Multimedia Appendix 3 [file ijmr_v6i2e15_app3.pdf]

Website	Affiliation	Identified in searches (n)	Range of search rank order
1	Government/hospital/clinic	4	2-9
2	Independent information website	3	3-14
3	Charity/private organization	4	1-11
4	Charity/private organization	3	6-7
5	Charity/private organization	4	5-11
6	Pharmaceutical company	1	13-13
7	Pharmaceutical company	2	8-11
8	Independent information website	1	8-8
9	Government/hospital/clinic	3	1-12
10	Government/hospital/clinic	3	2-19
