# Supplementary material for: Quality of Patient Information Websites About Congenital Heart Defects: Mixed-Methods Study of Perspectives Among Individuals With Experience of a Prenatal Diagnosis
Source: Interact J Med Res. 2017 Sep 12;6(2):e15. doi: 10.2196/ijmr.7844 (PMC5615220; doi:10.2196/ijmr.7844)
Supplement: Multimedia Appendix 4 [file ijmr_v6i2e15_app4.pdf]

Characteristic	Category	Continued pregnancy, n	Terminated pregnancy, n
**Age**	20-29 years	0	3
	30-39 years	1	1
	>39 years	3	1
**Born children**	None	0	3
	One	2	2
	Two	1	0
	More than three	1	0
**Highest education**	Senior high school	1	1
	University/College	3	4
